# Changing Population Size in McDonald–Kreitman Style Analyses: Artifactual Correlations and Adaptive Evolution between Humans and Chimpanzees

**DOI:** 10.1093/gbe/evac022

**Published:** 2022-02-10

**Authors:** Vivak Soni, Ana Filipa Moutinho, Adam Eyre-Walker

**Affiliations:** 1 School of Life Sciences, University of Sussex, Brighton, United Kingdom; 2 Department for Evolutionary Genetics, Max Planck Institute for Evolutionary Biology, Plon, Germany

**Keywords:** adaptive evolution, McDonald–Kreitman, human, chimpanzee

## Abstract

It is known that methods to estimate the rate of adaptive evolution, which are based on the McDonald–Kreitman test, can be biased by changes in effective population size. Here, we demonstrate theoretically that changes in population size can also generate an artifactual correlation between the rate of adaptive evolution and any factor that is correlated to the strength of selection acting against deleterious mutations. In this context, we have investigated whether several site-level factors influence the rate of adaptive evolution in the divergence of humans and chimpanzees, two species that have been inferred to have undergone population size contraction since they diverged. We find that the rate of adaptive evolution, relative to the rate of mutation, is higher for more exposed amino acids, lower for amino acid pairs that are more dissimilar in terms of their polarity, volume, and lower for amino acid pairs that are subject to stronger purifying selection, as measured by the ratio of the numbers of nonsynonymous to synonymous polymorphisms (*p*_N_/*p*_S_). All of these correlations are opposite to the artifactual correlations expected under contracting population size. We therefore conclude that these correlations are genuine.

Significance Understanding the factors that affect the rate of adaptive evolution is a major goal of evolutionary biology. We demonstrate that a commonly used method to estimate the rate of adaptive evolution can generate artifactual correlations between the rate of adaptive evolution and another variable when there has been a change in population size. We investigate a number of factors that might affect the rate of adaptive evolution in humans and chimpanzees, two species which have undergone a contraction in their effective population size since they diverged. We show that the rate of adaptive evolution is correlated to the residue’s relative solvent accessibility and the difference between amino acids in their physiochemical properties. We demonstrate that these correlations are not a consequence of contracting population size and are, therefore, genuine.

## Introduction

The rate of adaptive evolution in protein coding genes appears to vary at several different levels. First, the rate of adaptive evolution appears to differ between species. Some species, including many plants (Bustamante et al. 2002; Barrier et al. 2003; Schmid et al. 2005; Gossmann et al. 2010; also see Strasburg et al. 2009; Ingvarsson 2010; Slotte et al. 2010) and the yeasts of the genus Saccharomyces (Gossmann et al. 2012), appear to go through very little adaptive evolution, whilst many other species, including Drosophilids (Smith and Eyre-Walker 2002; Sawyer et al. 2003; Eyre-Walker and Keightley 2009; Haddrill et al. 2010), rodents (Halligan et al. 2010), and many multicellular animals (Galtier 2016; Rousselle et al. 2020), go through extensive adaptive evolution. The reasons for this variation remain unclear. It has been suggested that population size might be a factor; if adaptation is mutation limited, then one might expect species with large population sizes to adapt faster because they will generate the required mutations more rapidly. There is some evidence that species with large population sizes do undergo significantly faster adaptive evolution (Gossmann et al. 2012; Bataillon et al. 2015; Corbett-Detig et al. 2015; Rousselle et al. 2020; though see Galtier 2016). However, it is unclear whether species are ever limited by the supply of mutations—there appears to be abundant genetic variation for most traits—and even if they are limited, species with large population sizes are predicted to be closer to their optimal fitness, and hence they may not have to adapt as much as species with small population sizes (Lourenço et al. 2013).

At the next level down, there appears to be variation in the rate of adaptation between genes. This is in part due to differences in function, with genes involved in immunity (Clark et al. 2003; Chimpanzee Sequencing and Analysis Consortium 2005; Nielsen 2005; Sackton et al. 2007; Obbard et al. 2009), interaction with viruses (Enard et al. 2016), and male reproductive success (Pröschel et al. 2006; Haerty et al. 2007) having higher rates of adaptive evolution. Other factors also seem to be important, with the rate of adaptive evolution being higher in genes that recombine frequently (Presgraves 2005; Betancourt et al. 2009; Arguello et al. 2010; Mackay et al. 2012; Campos et al. 2014; Castellano et al. 2016; Moutinho et al. 2019), are located in regions of the genome with low functional DNA density (Castellano et al. 2016), have high mutation rates (Castellano et al. 2016), and reside on the X-chromosome (Langley et al. 2012; MacKay et al. 2012; Campos et al. 2014). Genes that have lower expression levels (Pál et al. 2001; Rocha and Danchin 2004; Subramanian and Kumar 2004; Wright et al. 2004; Lemos et al. 2005) or shorter coding sequence length (Zhang 2000; Lipman et al. 2002; Liao et al. 2006), also seem to have higher rates of adaptation.

Finally, there appears to be variation at the site level. This variation has been widely documented in site-level tests that compare the rate of nonsynonymous with synonymous substitution (Liberles et al. 2012). A number of factors seem to affect rates of adaptive evolution at the site level including protein secondary structure (Goldman et al. 1998; Guo et al. 2004; Choi et al. 2006) and the relative solvent accessibility (RSA) (Goldman et al. 1998; Choi et al. 2006; Lin et al. 2007; Franzosa and Xia 2009); RSA is a measure of how buried an amino acid is. In both *Drosophila* and *Arabidopsis* species, the rate of adaptive nonsynonymous substitution is positively correlated to the RSA (Moutinho et al. 2019). This suggests that amino acids on the surface of a protein have higher rates of adaptive substitution than those that are buried (Perutz et al. 1965; Overington et al. 1992; Goldman et al. 1998; Bustamante et al. 2000; Dean et al. 2002; Choi et al. 2006; Lin et al. 2007; Conant and Stadler 2009; Franzosa and Xia 2009; Ramsey et al. 2011). It has also been shown that amino acids that differ substantially in their physio-chemical properties, have lower rates of adaptive evolution than those that are more similar (Bergman and Eyre-Walker 2019; though see Gojobori et al. 2007; Chen et al. 2019). Finally, Bergman and Eyre-Walker (2019) also showed that amino acids pairs that are subject to high levels of negative selection have lower rates of adaptive substitution; they measured the level of negative selection using the ratio of the number of nonsynonymous to synonymous polymorphisms, *p*_N_/*p*_S_.

Many of the analyses discussed above used methods based on the McDonald–Kreitman test (McDonald and Kreitman 1991) to infer the rate of adaptive evolution (Boyko et al. 2008; Eyre-Walker and Keightley 2009; Galtier 2016). In these methods, evolution at sites subject to selection is compared with that at putatively neutral sites, using both polymorphism and divergence data; the site frequency spectrum (SFS), derived from the polymorphism data, is used to infer the distribution of fitness effects (DFE), and the DFE is then used to predict how many neutral or deleterious substitutions are expected at the selected sites between the two species. If more divergence is observed than expected, then adaptive evolution is inferred and quantified. It has however, been appreciated for a long time that population size change can lead to biased estimates of the rate of adaptive evolution (McDonald and Kreitman 1991; Eyre-Walker 2002). If the current effective population size, from which the polymorphism data are sampled, is larger than that during divergence, then rates of adaptive evolution will be overestimated (Eyre-Walker 2002). This is because slightly deleterious mutations, which might have been fixed during the divergence phase, no longer segregate because selection is more effective in the current larger population size. If the effective population size during the divergence phase is greater than the current, the rate of adaptive evolution tends to be underestimated.

Population size change might also affect the relationship between the estimated rate of adaptive evolution and a genomic variable. Here, we explore this possibility theoretically and show that population size change induces an apparent correlation between the rate of adaptive evolution and any genomic variable that is correlated to the mean strength of selection acting against deleterious mutations, even when no adaptive evolution has occurred. Hence, some of the correlations that have been observed between the rate of adaptive evolution and another variable could in fact be an artifact of population size change.

Humans and chimpanzees present an interesting case because both ancestral and current effective population sizes have been estimated, and these two species appear to have undergone a substantial decrease in their effective population size since they diverged (Hobolth et al. 2007; Burgess and Yang 2008; Prado-Martinez et al. 2013; Schrago 2014). Here, we consider whether the rate of adaptive evolution between humans and chimpanzees is correlated to several site level factors previously shown to be particularly important in other species—RSA and various measures of the difference between amino acids—and we investigate whether the apparent correlations could be an artifact of the decrease in effective population size. What we discover is the opposite. The decrease in effective population size is predicted to generate correlations that are contrary to those we observe, suggesting that the rate of adaptive evolution is genuinely correlated to a number of different genomic variables at the site level.

## Results

### Theory

It is well established that MK-type methods lead to biased estimates of the rate of adaptive evolution if the effective population size differs between the divergence and polymorphism phases (McDonald and Kreitman 1991; Eyre-Walker 2002). Could changes in effective population size also artifactually affect the relationship between the rate of adaptive evolution and another genomic variable, such as the difference in physico-chemical properties between two amino acids?

Let us assume that synonymous mutations are neutral and nonsynonymous mutations are neutral or subject to negative selection. The ratio of the nonsynonymous to synonymous substitution rates $\omega =\omega_{a}+\omega_{\mathrm{na}}$, where $\omega_{a}$ and $\omega_{\mathrm{na}}$ are the rate of adaptive and nonadaptive nonsynonymous substitution relative to the rate of synonymous substitution, which is an estimate of the mutation rate under this model. Hence,

(1) $$\omega_{a}=\omega -\omega_{\mathrm{na}}.$$

If we assume that all nonsynonymous are deleterious with effects drawn from a gamma distribution then:

(2) $$\omega \approx \frac{k}{{\left(N_{d}\overset{-}{s}\right)}^{\beta }}$$

(Welch et al. 2008, equation 23) where *N*_d_ is the effective population size during the divergence phase, *k* is a constant, β is the shape parameter of the gamma distribution, and $\overset{-}{s}$ is the mean absolute strength of selection acting against deleterious mutations.

We can also write a simple expression for $\omega_{\mathrm{na}}$. This is estimated in MK type approaches from polymorphism data, using the SFS at synonymous and nonsynonymous sites, to estimate the DFE at nonsynonymous sites. This DFE is then used to infer $\omega_{\mathrm{na}}$. Hence,

(3) $$\omega_{\mathrm{na}}=\frac{k}{{\left(N_{p}\overset{-}{s}\right)}^{\beta }},$$

where *N*_p_ is the effective population size pertaining to the polymorphism data.

Substituting equations (2) and (3) into (1), we get an expression for the estimated value of ω_a_,

(4) $$\omega_{a}^{'}=\frac{k}{{\left(N_{d}\overset{-}{s}\right)}^{\beta }}-\frac{k}{{\left(N_{p}\overset{-}{s}\right)}^{\beta }}=\frac{k\left({\left(N_{p}\overset{-}{s}\right)}^{\beta }-{\left(N_{d}\overset{-}{s}\right)}^{\beta }\right)}{{\left(N_{p}\overset{-}{s}\right)}^{\beta }{\left(N_{d}\overset{-}{s}\right)}^{\beta }}=\frac{k\left({\left(N_{p}/N_{d}\right)}^{\beta }-1\right)}{{\left(N_{p}\overset{-}{s}\right)}^{\beta .}}$$

From this equation, it is evident that $\omega_{a}^{'}$ > 0 if *N*_p_*>N*_d,_ and $\omega_{a}^{'}$ < 0 if *N*_p_*<N*_d_ as we expect. However, of more interest is the fact that the over- or under-estimation of *ω_a_* depends on $\overset{-}{s}$, the mean strength of selection acting against deleterious mutations. With population size expansion, we predict that *ω_a_* will be overestimated but that the magnitude of this overestimation will decrease as the mean strength of selection increases. Conversely, with population size contraction, *ω_a_* will be under-estimated and this underestimation will diminish as the mean strength of selection increases. Hence, under population size expansion, we expect a negative correlation between $\omega_{a}^{'}$ and any variable that is correlated to the mean absolute strength of selection acting against deleterious mutations and a positive correlation with population contraction, if there is no adaptive evolution.

If we note that,

(5) $$\frac{p_{N}}{p_{S}}=\frac{m}{{\left(N_{p}\overset{-}{s}\right)}^{\beta }}$$

(Welch et al. 2008, equation 26), where *m* is a constant which depends on how many chromosomes have been sampled and a scaling factor, then equation (4) can be rewritten as:

(6) $$\omega_{a}^{'}=k\left(\frac{{\left(N_{p}/N_{d}\right)}^{\beta }-1}{m}\right)\frac{p_{N}}{p_{S}}$$.

Hence, we expect $\omega_{a}^{'}$ to be positively and linearly correlated to *p*_N_/*p*_S_ if there was population size expansion and negatively correlated if there has been contraction and there is no adaptive evolution occurring.

An alternative measure of the rate of adaptive evolution is the proportion of substitutions that are fixed by positive selection. Under our model, this becomes:

(7) $$\alpha^{'}=\frac{\omega_{a}}{\omega }=1-{\left(\frac{N_{d}}{N_{p}}\right)}^{\beta }$$.

As expected, if *N*_p_*>N*_d_ then *α′* > 0, and if *N*_p_*<N*_d_ then *α′* < 0, however the magnitude of this bias is independent of the strength of selection acting upon deleterious mutations.

What do we expect if there has been adaptive evolution? Let the rate of adaptive evolution, relative to the mutation rate, potentially be a function of the mean strength of selection acting against deleterious mutations, $A\left(\overset{-}{s}\right).$ Then equation (2) becomes:

(8) $$\omega \approx \frac{k}{{\left(N_{d}\overset{-}{s}\right)}^{\beta }}+A\left(\overset{-}{s}\right),$$

which leads to a revision of equations (4) and (6):

(9) $$\omega_{a}^{`}=\frac{k\left({\left(N_{p}/N_{d}\right)}^{\beta }-1\right)}{{\left(N_{p}\overset{-}{s}\right)}^{\beta }}+A\left(\overset{-}{s}\right)\;\omega_{a}^{`}=\left(\frac{{\left(N_{p}/N_{d}\right)}^{\beta }-1}{m}\right)\frac{p_{N}}{p_{S}}+\;A\left(\overset{-}{s}\right).$$

Thus, if the rate of adaptive evolution is independent of the mean strength of selection acting against deleterious mutations, that is, $A\left(\overset{-}{s}\right)=a,$ then it is evident that our predictions, derived under the assumption of no adaptive evolution, hold—that is, population contraction will induce an artifactual positive correlation between $\omega_{a}^{'}$ and a variable that is correlated to the mean strength of selection against deleterious mutations. If the rate of adaptive evolution is correlated to the mean strength of selection, then this will tend to either increase or decrease the strength of the relationship.

### Data Analysis

Given the theoretical predictions derived above, is it of interest to examine patterns of adaptive evolution in the divergence of humans and chimpanzees, two species for which we know a substantial amount about their long-term demographic history; they appear to have undergone a population size contraction since they split. We have investigated whether several site-level factors affect the rate of adaptive and nonadaptive evolution in hominids—RSA, and measures of physico-chemical dissimilarity (volume and polarity) and an estimate of the average level of negative selection acting on mutations between two amino acids (*p*_N_/*p*_S_). We measure the rates of adaptive and nonadaptive evolution using the statistics *ω_a_* and *ω_na_*, which are respectively estimates of the rate of adaptive and nonadaptive evolution relative to the mutation rate. Both statistics were estimated using an extension of the McDonald–Kreitman method (McDonald and Kreitman 1991) taking into account the influence of slightly deleterious mutations. We use the method implemented in GRAPES (Galtier 2016), which is a maximum likelihood implementation of the second method proposed by Eyre-Walker and Keightley (2009).

### Relative Solvent Accessibility

**Fig. 1.**
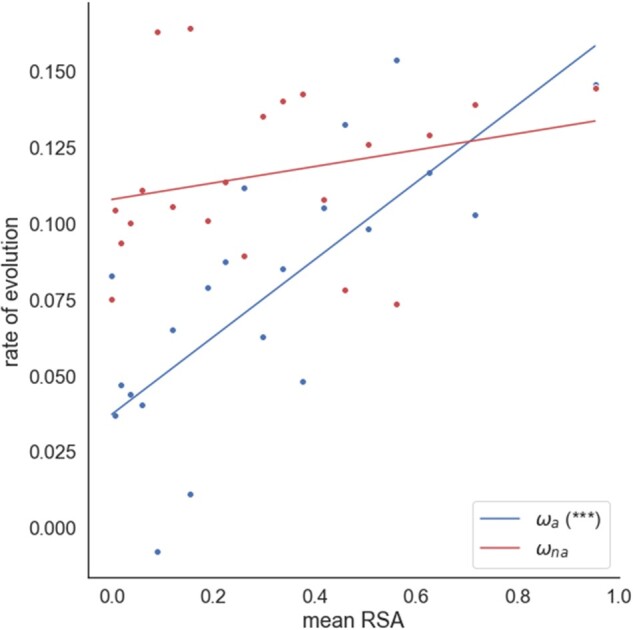
Estimates of ω_a_ and ω_na_ plotted against mean relative solvent accessibility. Data binned into 20 RSA bins of roughly equal number of sites. For each analysis, a weighted linear regression is fitted to the data. The respective significance of each correlation is shown in the plot legend (*P < 0.05; **P < 0.01; ***P < 0.001; “.” 0.05 ≤ *P* < 0.10). Regression is weighted by the reciprocal of the variance for each estimate of *ω_a_* and *ω_na_*, which were estimated by bootstrapping the data by gene 100 times for each data point.

Previous studies have shown that amino acid residues at the surface of proteins evolve faster than those at the core (Goldman et al. 1998; Choi et al. 2006; Lin et al. 2007; Franzosa and Xia 2009). These studies do not distinguish whether this higher substitution rate is due to reduced selective constraints on exposed residues or an increased rate of adaptive substitutions (or both). Moutinho et al. (2019) disentangled these effects by estimating both the rates of adaptive and nonadaptive evolution across several RSA categories in *Drosophila* and *Arabidopsis*, finding positive correlations between RSA and the rates of both adaptive and nonadaptive substitution. Their findings suggest that both reduced negative selection and a higher rate of adaptive evolution operate on more exposed residues. We find a significant correlation between the rate of adaptive evolution and RSA (*r* = 0.486, *P* < 0.001) when we use a weighting by the reciprocal of the variance of the rate of adaptive or nonadaptive evolution. However, the correlation with the rate of nonadaptive evolution is nonsignificant (*r* = 0.001, *P* = 0.324) (fig. 1). To check that our grouping scheme did not adversely affect our results, we repeated our analysis randomly allocating genes to RSA bins, estimating the rate of adaptive evolution and re-estimating the slope of the relationship between *ω_a_* and *ω_na_*; in none of 100 randomized data sets did we see a correlation as strong as that observed for *ω_a_* in the real data.

### Amino Acid Dissimilarity

**Fig. 2.**
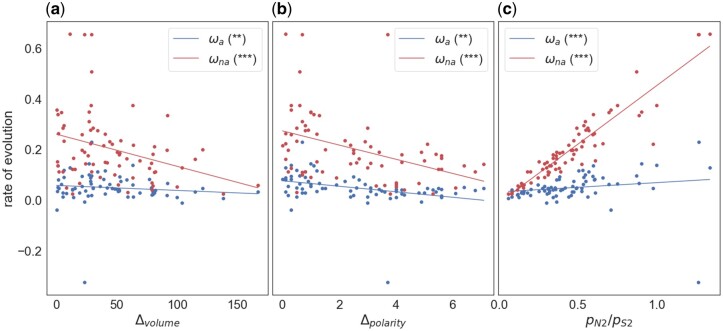
The adaptive and nonadaptive substitution rate plotted against the difference in (*a*) volume, (*b*) polarity, and (*c*) the ratio of nonsynonymous to synonymous polymorphisms, *p*_N2_/*p*_S2_, for 75 pairs of amino acids In (*c*), the polymorphisms are split by sampling from a hypergeometric distribution, with one set used to calculate rates of adaptive and nonadaptive substitution and the other to estimate the polymorphism statistics. A weighted linear regression is fitted to the data, weighted by the variance of each estimate. The respective significance of each correlation is shown in the legend (*P < 0.05; **P < 0.01; ***P < 0.001; “.” 0.05 ≤ *P* < 0.10).

To investigate whether the rates of adaptive and nonadaptive evolution are affected by amino acid dissimilarity, we estimated *ω_a_* and *ω_na_* between all 75 pairs of amino acids that are separated by a single mutational step in hominids. Bergman and Eyre-Walker (2019) found negative correlations between measures of amino acid dissimilarity (differences in volume and polarity) and *ω_a_* between *Drosophila* species. We find that the rate of adaptive substitution is significantly negatively correlated to Δvolume (*r*=−0.290, *P* = 0.018) and Δpolarity (*r* =−0.269, *P* = 0.027) (fig. 2*a* and *b*) when we fit a weighted linear regression to the data, suggesting that the rate of adaptive evolution is higher between more physiochemically similar amino acids. Similar negative correlations are observed for the rate of nonadaptive evolution (Δvolume: *r*=−0.545, *P* < 0.001; Δpolarity: *r*=−0.170, *P* < 0.001); these correlations remain highly significant (*P* < 0.001 in both cases) even if the four datapoints in the top left-hand corner are removed. The slopes are significantly steeper for *ω_na_* than *ω_a_* (table 1); however, this appears to be simply because rates of nonadaptive evolution are greater than rates of adaptive evolution; when we divide *ω_a_* and *ω_na_* by their means, the slopes are not significantly different (table 1).

**Table 1:** The Slope of the Relationship between *ω_a_* and *ω_na_* and the ΔVolume and ΔPolarity; Rescaled Values Are Where *ω_a_* and *ω_na_* Have Been Divided by Their Means

		ω_a_		ω_na_		
Statistic	Rescaled	Slope	SE	Slope	SE	Sig.
ΔVolume	No	−0.00027	0.000098	−0.00009	0.00026	0.012
ΔPolarity	No	−0.0064	0.0020	−0.0020	0.0054	0.010
*p*_N_/*p*_S_	No	0.060	0.020	0.41	0.021	0.000
ΔVolume	Yes	−0.0054	0.0020	−0.0020t	0.0013	ns
ΔPolarity	Yes	−0.13	0.042	−0.042	0.027	ns
*p*_N_/*p*_S_	Yes	1.3	0.41	2.0	0.10	ns

Note.—Significance was measured using an analysis of variance.

The difference in polarity and volume are not significantly correlated to each other (*r* = 0.122, *P* = 0.258), so it seems likely that both Δvolume and Δpolarity have an influence over the rate of adaptive and nonadaptive evolution. A multiple regression confirms this for *ω_na_* with both factors being highly significant and of similar influence, as judged by standardized regression coefficients (Δvolume *b*_s_=−0.29, *P* = 0.015; Δpolarity *b*_s_=−0.31, *P* = 0.008). For *ω_a_*, only Δpolarity is significant (Δvolume *b*_s_=−0.19, *P* = 0.14; Δpolarity *b*_s_=−0.27, *P* = 0.036); the loss of significance for Δvolume is probably due to a loss of power due to lack of data; in multiple regression, we are effectively holding one variable constant and testing whether the other remains significant.

Volume and polarity reflect only two of the multiple ways in which amino acids differ. As an alternative measure of amino acid dissimilarity, Bergman and Eyre-Walker (2019) suggest using the ratio of nonsynonymous to synonymous polymorphism; *p*_N_/*p*_S_ is expected to decrease as the strength of selection against deleterious mutations increases. We find that hominids are consistent with this expectation as *p*_N_/*p*_S_ is negatively correlated with both amino acid volume difference (*r*=−0.456, *P* < 0.001) and polarity difference (*r* =−0.269, *P* = 0.047). Polymorphism data are used to estimate both the rates of adaptive and nonadaptive substitution, meaning that *p*_N_/*p*_S_ is not statistically independent of either measure. To account for this source of sampling error, we follow the method of Bergman and Eyre-Walker (2019), resampling the SFS using a hypergeometric distribution to generate two independent spectra. One of these is used to estimate *p*_N_/*p*_S_ (referred to as *p*_N2_/*p*_S2_) and the other is used to estimate *ω_a_* and *ω_na_*, therefore removing the nonindependence between *p*_N_/*p*_S_ and *ω_a_* and *ω_na_*. We find that *ω_a_* is positively correlated to *p*_N1_/*p*_S1_ (*r* = 0.419, *P* < 0.001) in hominids, consistent with previous findings in *Drosophila* (Bergman and Eyre-Walker 2019). Consistent with our physico-chemical dissimilarity correlations, *ω_na_* also shows a positive correlation with *p*_N1_/*p*_S1_. The correlation is stronger and the slope steeper than we see for *ω_a_* (*r* = 0.882, *P* < 0.001) (fig. 2*c* andtable 1); however, if we divide *ω_a_* and *ω_na_* by their means, we find that the slopes are no longer significantly different (table 1).

It is possible that the correlations between *ω_a_* and *ω_na_* and various site level factors are interrelated; for example, the positive correlation between ω_a_ and RSA might be due to amino acids that are found exposed on the surface of proteins being one mutational step closer to similar amino acids. However, there is no correlation between the average RSA of an amino acid and the average difference in volume or polarity to its one mutation step neighbors (RSA-Δvolume: *r* =−0.171, *P* = 0.471; RSA-Δpolarity: *r* = 0.059, *P* = 0.803—supplementary fig. S1, Supplementary Material online).

### Biased Gene Conversion

Biased gene conversion can potentially impact estimates of the rate of adaptive evolution, since it increases the fixation probability of Weak (W) to Strong (S) alleles relative to S > W neutral alleles, more than it increases levels of W > S polymorphisms relative to S > W polymorphisms; a problem exacerbated by differences in base composition between synonymous and nonsynonymous sites (Galtier and Duret 2007; Berglund et al. 2009; Ratnakumar et al. 2010; Rousselle et al. 2020). To investigate whether the correlation between the rates of adaptive and nonadaptive evolution and our measures of amino acid dissimilarity are due to BGC, we restricted the analysis to polymorphisms and substitutions that involve nucleotide changes that are unaffected by BGC—that is, A<>T and G<>C changes. This reduces our data set substantially by removing 80% of our substitutions and polymorphisms and reducing the amino acid analysis to just 12 amino acid pairs. However, we find that the correlations between *ω_a_*, RSA, Δvolume, and *p*_N_/*p*_S_ all remain significant with only the correlation to Δpolarity becoming nonsignificant (RSA: *r* = 0.260, *P* < 0.05; Δvolume: *r* =−0.576, *P* < 0.01; Δpolarity: *r* =−0.166, *P* < 0.1; *p*_N2_/*p*_S2_: *r* = 0.796, *P* < 0.001); the correlations between the rate of nonadaptive evolution, *ω_na_*, and Δvolume and *p*_N2_/*p*_S2_ remain significant (RSA: *r* = 0.011, *P* = 0.370; Δvolume: *r* = 0.513, *P* < 0.01; Δpolarity: *r* = 0.115, *P* = 0.150; *p*_N2_/*p*_S2_: *r* = 0.804, *P* < 0.001).

### Are the Correlations Artifactual?

**Fig. 3.**
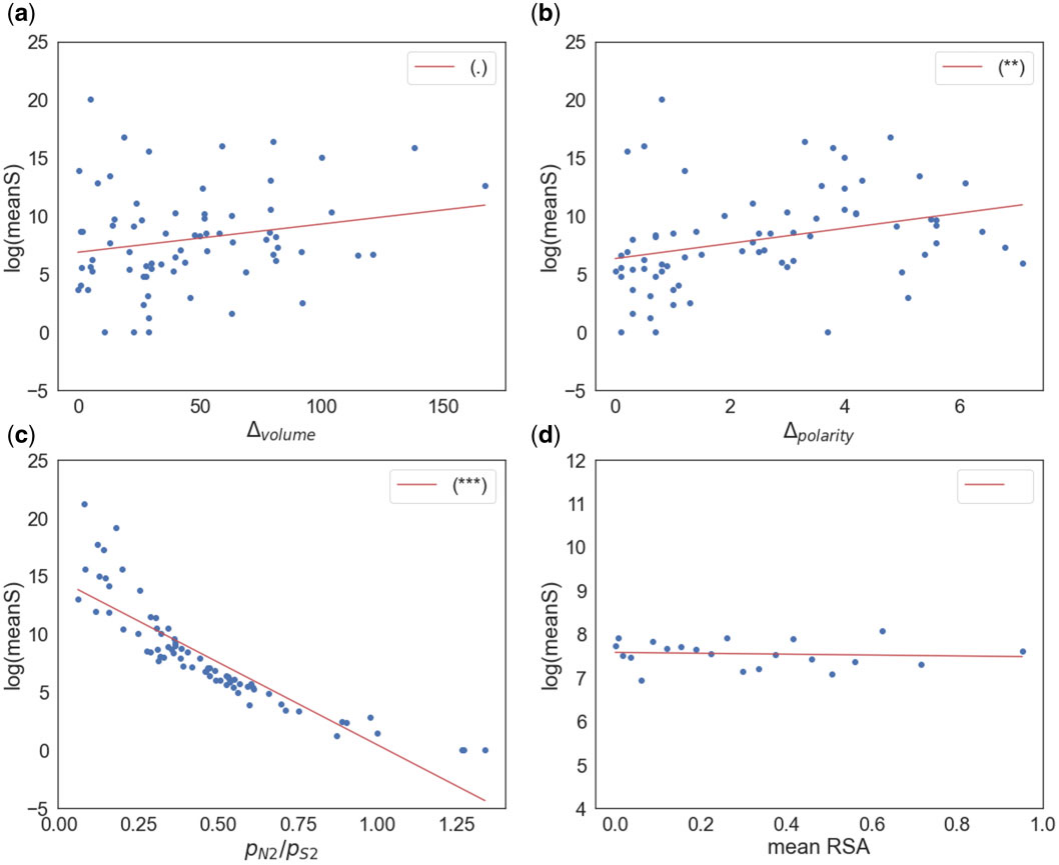
Log(mean*S*) plotted against (*a*) volume difference, (*b*) polarity difference, (*c*) *p*_N2_/*p*_S2_, (*d*) mean RSA. The respective significance of each correlation is shown in the plot legend, (*P < 0.05; **P < 0.01; ***P < 0.001; “.” 0.05 ≤ *P* < 0.10) based on an unweighted regression fit to the data.

In summary, we have shown that *ω_a_* is significantly positively correlated to RSA and *p*_N_/*p*_S_, and negatively correlated to the difference in polarity and volume. Could these correlations be explained as an artifact of population size contraction? The method we have used to estimate $\omega_{a}$ generates an estimate of the mean absolute strength of selection acting against deleterious mutations. We find that log(|$\overset{-}{s}$|) is positively correlated to Δvolume (*r* = 0.205, *P* = 0.08) and Δpolarity (*r* = 0.310, *P* = 0.008) and significantly negatively correlated to *p*_N_/*p*_S_ (*r* =−0.880, *P* < 0.001) but there is no correlation with RSA (*r* =−0.088, *P* = 0.704) (fig. 3). Thus, if there was no adaptive evolution, or the rate of adaptive evolution was independent of the variable being investigated (e.g., the difference in polarity), then we would expect $\omega_{a}$ to be positively correlated to the difference in volume and polarity, and negatively correlated to *p*_N_/*p*_S_. In fact, we observe the opposite pattern in each case suggesting that these correlations are not an artifact of population size contraction, but are genuine.

### Comparison to *Drosophila*

It is of interest to ask how the slopes of the relationships between *ω_a_* and each factor compares with those previously estimated in *Drosophila* species (Bergman and Eyre-Walker 2019; Moutinho et al. 2019). We find that the slope is not significantly different for RSA, Δvolume, and Δpolarity. However, the slope between ω_a_ and *p*_N_/*p*_S_ is much steeper in Drosophilids than in hominids (table 2). This might be because of population contraction. For each genomic variable, population size contraction is expected to reduce the slope of the relationship between ω_a_ and the factor in the human–chimp comparison, except for RSA which is not correlated to the mean strength of selection. However, the relationship between log(|$\overset{-}{s}$|) and *p*_N_/*p*_S_ is much stronger and steeper than for the other variables; if we standardize the variables by subtracting the mean and dividing by the standard deviation the slopes between log(|$\overset{-}{s}$|) and each factor are: RSA=−0.101, Δvolume *b* = 0.862, Δpolarity, *b* = 1.30, *p*_N_/*p*_S_=−3.90. Hence, we might expect population contraction to have a disproportionate effect on the relationship between ω_a_ and *p*_N_/*p*_S._

**Table 2:** Slopes of the Regressions between *ω_a_* and Measures of Amino Acid Dissimilarity in Hominid and *Drosophila* Data Sets

		Hominids (This Analysis)		Drosophila (Bergman and Eyre-Walker 2019)		Sig.
Data Set	Independent Variable	Slope	SE of Slope	Slope	SE of Slope	
Original	RSA	0.13	0.029	0.078	0.0065	ns
Original	ΔVol	−0.00026	0.00010	−0.00027	0.000061	ns
Original	ΔPol	−0.0064	0.0020	−0.0047	0.0011	ns
Original	*p*_N_/*p*_S_	0.061	0.019	0.29	0.029	<0.001
Rescaled	RSA	1.6	0.36	1.6	0.13	ns
Rescaled	ΔVol	−0.0054	0.0020	−0.011	0.0024	ns
Rescaled	ΔPol	−0.13	0.042	−0.18	0.041	ns
Rescaled	*p*_N_/*p*_S_	1.3	0.40	11	1.1	<0.001

Note.—In the rescaled analyses, the ω_a_ values have been divided by their mean. The slopes for the *Drosophila* analysis were obtained from the results supplied by Bergman and Eyre-Walker (2019).

## Discussion

One of the main weaknesses of the methods that estimate the rate of adaptive evolution using a McDonald–Kreitman type approach is their sensitivity to changes in the effective population size. With an expansion in population size, these methods overestimate the rate of adaptive evolution, and with a contraction, they underestimate it (Eyre-Walker 2002). Here, we demonstrate an additional problem. MK-style methods are also susceptible to producing artifactual correlations between the rate of adaptive evolution, scaled relative to the mutation rate, and another variable, such as amino acid dissimilarity, if that variable is correlated to the mean absolute strength of selection acting against deleterious mutations. This then might call into question previous correlations of this type. For example, it has been observed that *p*_N_/*p*_S_, for pairs of amino acids separated by one mutational step, is negatively correlated to the mean strength of selection in Drosophilids (Bergman and Eyre-Walker 2019). Hence, the positive correlation between *ω_a_* and *p*_N_/*p*_S_ across pairs of amino acids in these species (Bergman and Eyre-Walker 2019) could simply be an artifact of population size expansion, although there is no evidence that population size expansion has affected the species involved. There might be no adaptive evolution, and if there is adaptive evolution, its rate may not be correlated to *p*_N_/*p*_S_. In future, attempts should be made to estimate the mean strength of selection acting against deleterious mutations and investigate whether this is correlated to the factor in question; for example, if we are investigating whether the rate of adaptive evolution is correlated to the rate of recombination, we should investigate whether the mean strength of selection is correlated to the rate of recombination. If it is, then we should be cautious about interpreting our results unless we know something about the demographic history of the species.

Humans and chimpanzees are potentially useful because both their ancestral and current effective population sizes have been estimated; analyses suggest that the human–chimp ancestral population size was considerably larger than the current effective population size of either species (Hobolth et al. 2007; Burgess and Yang 2008; Prado-Martinez et al. 2013; Schrago 2014). Given the observed correlations between each factor and the mean strength of selection, we predict, under population size contraction, that the correlations should be opposite to those observed. Hence, it seems that the correlations between ω_a_ and RSA, Δvolume, Δpolarity, and *p*_N_/*p*_S_ are all genuine, in hominids at least, and this lends support to the notion that similar correlations in *Drosophila* and *Arabidopsis* species are also real. However, some caution should be exercised because although we know something about the effective population of the ancestral and current populations of humans and chimpanzees, we know little about the population size in between these timepoints. For example, the ancestral population may have contracted after the species diverged and subsequently re-expanded toward the present. Under this scenario, the effective population during the divergence phase could have been lower than that during the polymorphism phase. Furthermore, changes in *N*_e_ affect neutral and selected mutations differently (Otto and Whitlock 1997). Since, human population sizes have increased dramatically recently, the effective population size for deleterious genetic variation is greater than for neutral variation, because the deleterious mutations are younger on average. It is therefore possible that the slightly deleterious genetic variation, which can potentially bias MK-style methods, has not experienced a smaller *N*_e_ during the polymorphism relative to the divergence phase. However, this seems unlikely; the current *N*_e_ for neutral variation is estimated to be between 5- and 10-fold lower than the population size of human–chimpanzee ancestor (Hobolth et al. 2007; Burgess and Yang 2008; Prado-Martinez et al. 2013; Schrago 2014), and the mutations that are most likely to affect the method to estimate the rate of adaptive evolution are weakly selected.

Population contraction leads to an underestimate of the rate of adaptive evolution when using MK-style methods (McDonald and Kreitman 1991; Eyre-Walker 2002). As a consequence, Zhen et al. (2021) have argued that the rate of adaptive evolution between humans and chimpanzees has been underestimated, and that they have undergone higher rates of adaptive evolution than *Drosophila* species. In fact, the average of $\omega_{a}$ across amino acid pairs is significantly higher in hominids than *Drosophila* (hominids, mean $\omega_{a}$ = 0.0488 [SE = 0.0072]; *Drosophila* mean $\omega_{a}$ = 0.0258 [SE = 0.0024]; *t*-test *t* = 3.01, *P* < 0.001), so hominids seem to be adapting faster relative to the mutation rate even without taking into account population contraction. What is perhaps surprising is that $\omega_{a}$ is not negative even when we correlate it against factors that appear to influence it. The observed value of $\omega_{a}$ is expected to be equal to:

(10) $$\omega_{a}\left(\mathrm{obs}\right)=\omega_{a}\left(\mathrm{true}\right)+\omega_{a}(\mathrm{predicted}),$$

where $\omega_{a}\left(\mathrm{true}\right)$ is the true value, and $\omega_{a}(\mathrm{predicted})$ is the value predicted in the absence of adaptive evolution from equations (4) or (6); that is, it is the bias in the estimate due to the differences in the effective population size between the divergence and polymorphism phases. For example, $\omega_{a}$ is positively correlated to RSA, however, even those sites with very low RSA, have a positive estimate of $\omega_{a}$.This seems surprising and suggests that adaptive evolution is more prevalent than we thought in hominids. However, predicting how much is difficult because we do not know how the effective population size has changed during the divergence of humans and chimpanzees.

We confirm the findings of Moutinho et al. (2019) with respect to RSA—more exposed amino acid residues have higher rates of adaptive evolution. Moutinho et al. (2019) also showed that the rate of nonadaptive evolution is positively correlated to RSA. These observations are consistent with two models of evolution; either the fitness landscape is relatively flat for more exposed residues, or the mutational steps are relatively small. It is difficult to differentiate between these models.

We also confirm the results of Bergman and Eyre-Walker (2019)—rates of adaptive and nonadaptive evolution are lower between more dissimilar amino acids. It seems likely that these correlations are due to the mutational steps being smaller and hence that adaptive evolution proceeds via small steps in this component of evolution. Chen et al. (2019) apparently came to a different conclusion but their analysis largely focused on a statistic that is related to the proportion of substitutions that are adaptive, and hence conflates the pattern of adaptive and nonadaptive evolution. In fact, consistent with their findings and those of Bergman and Eyre-Walker (2019), we find the proportion of substitutions that are adaptive is uncorrelated to either the difference in volume or polarity (Δvolume: *r* =−0.012, *P* = 0.707; Δpolarity: *r* = 0.0003, *P* = 0.314); however, the proportion is significantly positively correlated to both *p*_N1_/*p*_S1_ (*r* = 0.20, *P* = 0.046) and RSA (*r* = 0.44, *P* = 0.027).

In summary, we demonstrate that population size change can lead to an artifactual correlation between a measure of adaptive evolution and any variable related to the mean strength of selection against deleterious mutations. Our analysis in hominids suggests that there are genuine negative correlations between $\omega_{a}$ and amino acid dissimilarity and positive correlations between $\omega_{a}$ and RSA and a measure of negative selection acting on mutations between pairs of amino acid mutations, because under population size contraction we would expect the opposite.

## Materials and Methods

### Data

We obtained gene sequences from Ensembl’s biomart (Yates et al. 2019) for the human GRCh38 genome build and for the Pan_tro_3.0 chimpanzee genome build. Orthologous genes were aligned using MUSCLE (Edgar 2004). After filtering out genes with gaps that were not multiples of three, we were left with 16,344 pairwise alignments. Numbers of synonymous and nonsynonymous substitutions per site were obtained using PAML’s codeml (Yang 2007) program. We used polymorphism data from the African superpopulation of the 1000 genomes data set (1000 Genomes Project Consortium 2015) to construct our SFS, with rates of adaptive and nonadaptive evolution estimated using Grapes (Galtier 2016), under the “GammaZero” model. We chose African data because the African population is thought to have undergone less complex demographic changes then other human populations (Gutenkunst et al. 2009; Gravel et al. 2011). We fitted a weighted regression to our estimates of the rate of evolution, weighting by the reciprocal of the variance for each estimate of ω_a_ and ω_na_. The confidence interval and variance on our estimates of ω_a_ and ω_na_ were obtained by bootstrapping the data set by gene 100 times.

### RSA Analysis

In order to obtain structural information for each protein sequence, we ran BlastP (Schäffer et al. 2001) to assign each protein sequence to a PDB structure, and respective chain, by using the “pdbaa” library and an *E*-value threshold of 10^−10^. In instances of multiple matches, the match with the lowest *E*-value was kept. The corresponding PDB structures were further processed to only keep the corresponding chain per polymer. PDB manipulation and analysis were carried on using the R package “bio3d” (Grant et al. 2006). Values for solvent accessibility (SA) per residue were obtained using the “dssp” program with default options. To map SA values to each residue of the protein sequence a pairwise alignment between each protein and the respective PDB sequence was performed with MAFFT, allowing gaps in both sequences in order to increase the block size of sites aligned. The final data set comprised a total of 7,984,041 sites with SA information. We computed the RSA by dividing SA by the amino-acid’s SA area (Tien et al. 2013), giving us a final data set of 3,505,615 sites for which we have RSA information. These sites were grouped into 20 RSA bins of roughly equal size in terms of the number of sites, with rates of adaptive and nonadaptive evolution estimated for each bin. These rates were correlated with the mean RSA of each bin.

### Amino Acid Dissimilarity Analysis

For the amino acid dissimilarity analysis, we followed the methodology outlined in Bergman and Eyre-Walker (2019), with amino acid polarity and volume scores taken from data available in the AAindex1 database (Kawashima et al. 2007). We compared the SFS for a particular amino acid pair with synonymous data from 4-fold degenerate codons separated by the same mutational step. For example, alanine and glycine are separated by a single nucleotide change (C<>G at second position). Therefore, we used the SFS and divergence for all 4-fold degenerate codons separated by a single C<>G mutational step in estimating *ω_a_* and *ω_na_*. For amino acids separated by more than one mutational step (e.g., a C<>G or an A<>T mutational step), we used a weighted average SFS from the SFSs for the mutational types at 4-fold sites, weighting by the frequency of the respective codons as in Bergman and Eyre-Walker (2019).

For the analysis involving *p*_N_/*p*_S_, we used a hypergeometric distribution to resample the SFS, and generate two SFSs, one used to estimate rates of adaptive and nonadaptive evolution, and one used to estimate *p*_N_/*p*_S_.

## Supplementary Material

Supplementary data are available at *Genome Biology and Evolution* online.

## Data Availability

The analysis uses publicly available data. Scripts used to process the data are available at https://github.com/vivaksoni/site_level_factors_affecting_rates_of_evolution_in_hominids.

## Supplementary Material

evac022_Supplementary_Data
