# Supplementary material for: Changing Population Size in McDonald–Kreitman Style Analyses: Artifactual Correlations and Adaptive Evolution between Humans and Chimpanzees
Source: Genome Biol Evol. 2022 Feb 10;14(2):evac022. doi: 10.1093/gbe/evac022 (PMC8877167; doi:10.1093/gbe/evac022)
Supplement: evac022_Supplementary_Data [file evac022_supplementary_data.docx]

**Supplementary Material**


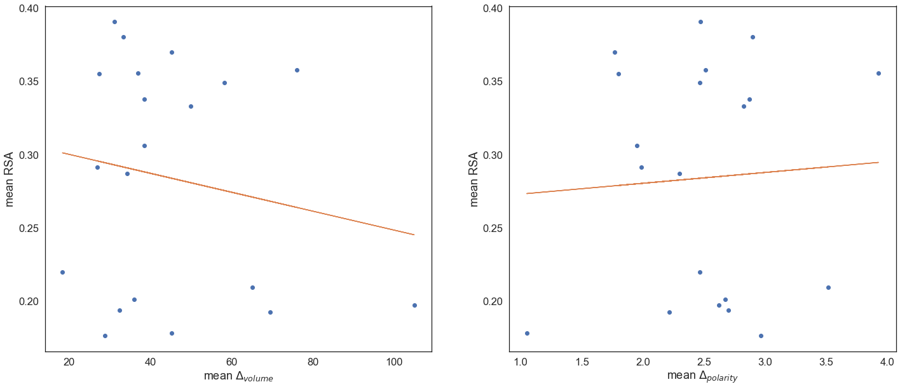


**Supplementary figure S1:** Average RSA of an amino acid and the average difference in volume or polarity to its one mutation step neighbours.


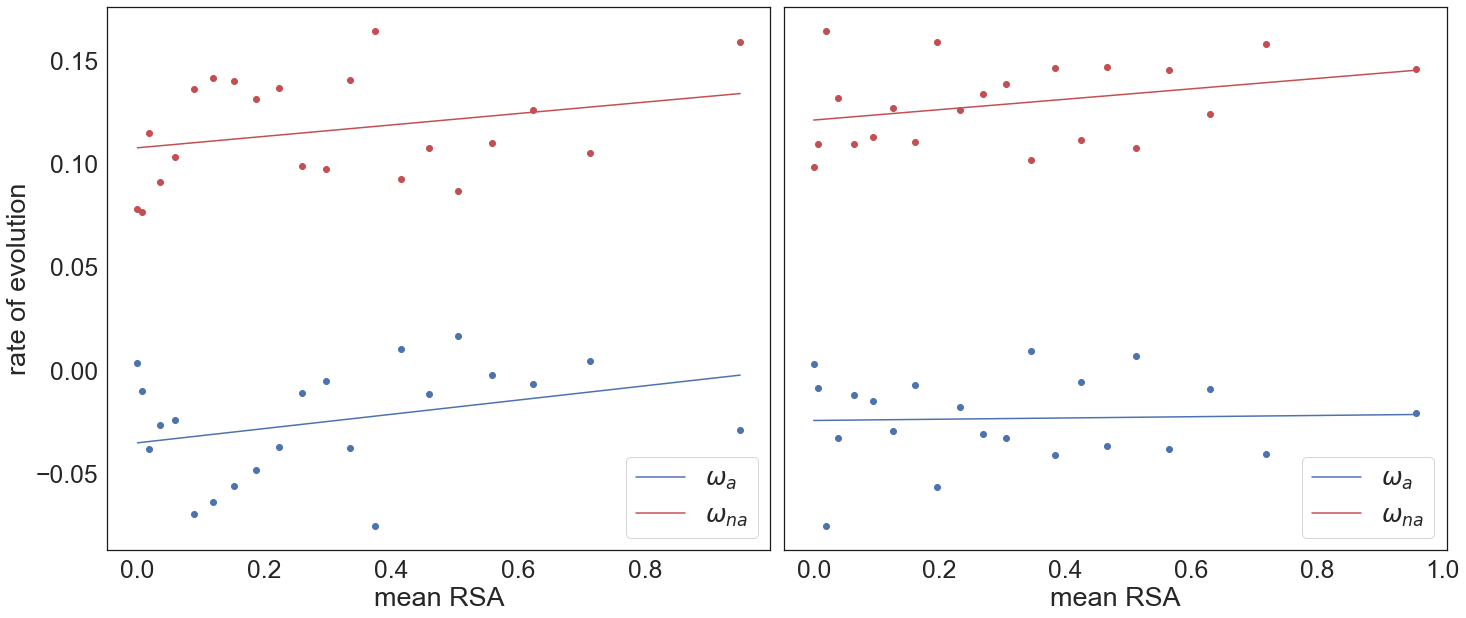


**Supplementary Figure S2:** Estimates of ω_a_ and ω_na_ plotted against mean relative solvent accessibility, controlling for volume difference (left) and polarity difference (right). Data binned into 20 RSA bins of roughly equal size. For each analysis, a weighted linear regression is fitted to the data. The respective significance of each correlation is shown in the plot legend, (*P < 0.05; **P < 0.01; ***P < 0.001; “.” 0.05 ≤ P < 0.10) for ω_a_ and ω_na_). Regression is weighted by the reciprocal of the variance for each estimate of ω_a_ and ω_na_, which were estimated by bootstrapping the data by gene 100 times for each data point.


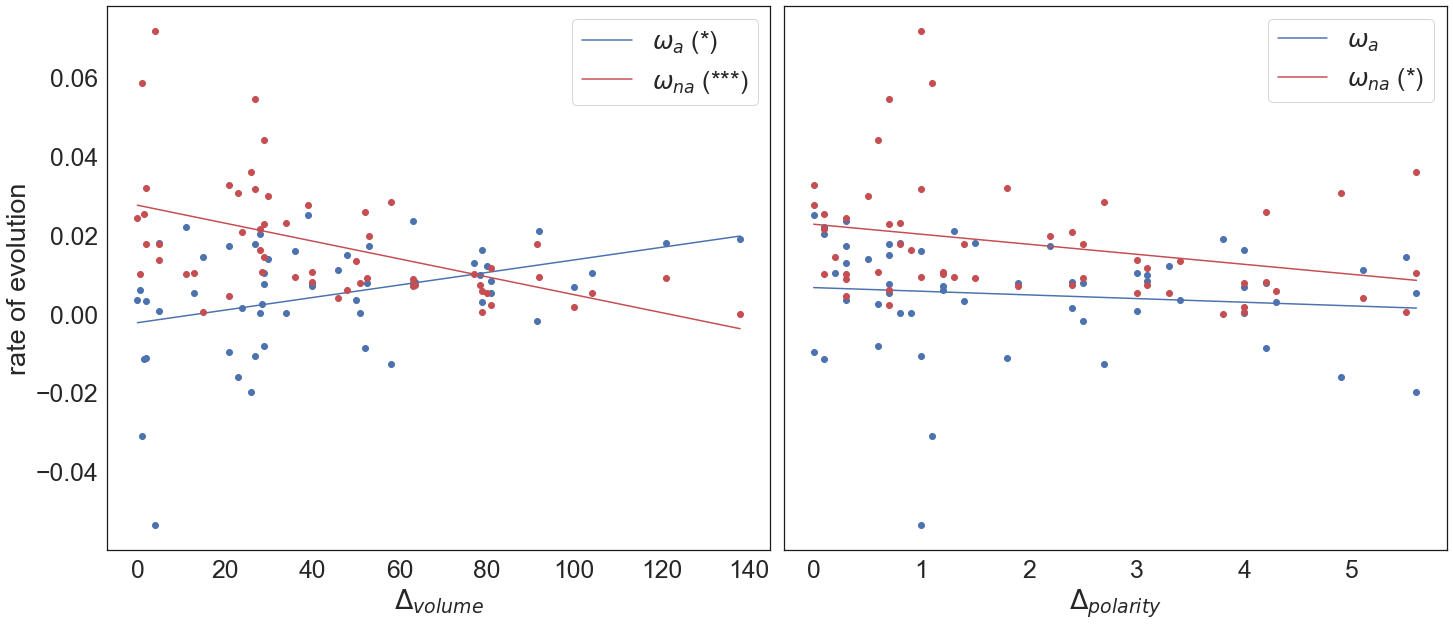


**Supplementary** **Figure S3:** The adaptive and non-adaptive substitution rate plotted against the difference in a) volume, b) polarity, controlling for relative solvent accessibility. A weighted linear regression is fitted to the data, weighted by the variance of each estimate. The respective significance of each correlation is shown in the legend, (*P < 0.05; **P < 0.01; ***P < 0.001; “.” 0.05 ≤ P < 0.10).
